# Attention-based solubility prediction of polysulfide and electrolyte analysis for lithium–sulfur batteries

**DOI:** 10.1038/s41598-023-47154-0

**Published:** 2023-11-27

**Authors:** Jaewan Lee, Hongjun Yang, Changyoung Park, Seong-Hyo Park, Eunji Jang, Hobeom Kwack, Chang Hoon Lee, Chang-ik Song, Young Cheol Choi, Sehui Han, Honglak Lee

**Affiliations:** 1 LG AI Research, ISC, 30, Magokjungang 10-ro, Gangseo-gu Seoul, 07796 Republic of Korea; 2 LG Energy Solution, LTD., LG Science Park E5, 30, Magokjungang 10-ro, Gangseo-gu Seoul, 07796 Republic of Korea

**Keywords:** Materials chemistry, Materials for energy and catalysis, Computational science

## Abstract

During the continuous charge and discharge process in lithium-sulfur batteries, one of the next-generation batteries, polysulfides are generated in the battery’s electrolyte, and impact its performance in terms of power and capacity by involving the process. The amount of polysulfides in the electrolyte could be estimated by the change of the Gibbs free energy of the electrolyte, $\Delta _{mix}\textrm{G}$ in the presence of polysulfide. However, obtaining $\Delta _{mix}\textrm{G}$ of the diverse mixtures of components in the electrolyte is a complex and expensive task that shows itself as a bottleneck in optimization of electrolytes. In this work, we present a machine-learning approach for predicting $\Delta _{mix}\textrm{G}$ of electrolytes. The proposed architecture utilizes (1) an attention-based model (Attentive FP), a contrastive learning model (MolCLR) or morgan fingerprints to represent chemical components, and (2) transformers to account for the interactions between chemicals in the electrolyte. This architecture was not only capable of predicting electrolyte properties, including those of chemicals not used during training, but also providing insights into chemical interactions within electrolytes. It revealed that interactions with other chemicals relate to the logP and molecular weight of the chemicals.

## Introduction

Lithium sulfur (LiS) batteries have been rapidly receiving attention as the next-generation secondary battery that can surpass lithium-ion batteries in perspective of cell capacity, lightness, and cost^1–4^. During the charge/discharge process of the battery, lithium polysulfides (LiPS), ${\hbox {Li}_{2}\hbox {S}_{\textrm{x}}}$ $(1 \le x \le 8)$, are formed from sulfur, cyclo-S8, by reacting with Li-ions at the cathode, and keep changing the formations. As a result, the concentration of LiPS in the electrolyte is a crucial factor in designing electrolytes. While it can be controlled based on solubility, it is challenging to measure all electrolytes experimentally because the electrolyte is typically a mixture of four to six chemicals with varying proportions. Instead, the Gibbs free energy of mixing, $\Delta _{mix}\textrm{G}$, that researchers have been used to understand mechanism of the LiPS could be used to estimate the solubility. For instance, if $\Delta _{mix}\textrm{G}$ of a chemical is less than 0, it indicates that the chemical is inclined to dissolve in the electrolyte.

Machine learning has been extensively adopted for various applications, ranging from predicting material properties to designing new materials. Common examples include predicting properties of small molecules such as estimating the binding affinity of a molecule to proteins^5,6^, physical or chemical properties of organic molecules or inorganic materials^7,8^. In real-world applications like pharmaceuticals and battery electrolytes, mixtures are more prevalent than single substances. However, most existing machine learning research has focused on single substances. Recently, Wang et al.^9^ made a progress in this area by introducing an attention-based model specifically designed to predict the properties of inorganic mixtures. The attention mechanism has been utilized not only in language and vision tasks but also in predicting chemical properties. Attention mechanisms offer both precise predictions and the ability to provide explanations for the results. For example, Attentive FP^10^, which utilized graph attention mechanisms, achieved state-of-the-art performance in predicting chemical properties and remains one of the best models in the field.

Here, we developed a model using molecular representations and the attention mechanism to predict $\Delta _{mix}\textrm{G}$ of LiPS in electrolytes, mixtures of chemicals. This model enabled us to predict electrolytes even containing chemicals not used during training, and to analyze the attention scores to interpret the origin of physical properties driving the interactions between electrolyte chemicals. Key contributions of our works include following:

- The highly accurate $\Delta _{mix}\textrm{G}$  prediction model for electrolytes was developed. Also, it could be applicable to electrolyte containing chemicals not used during training.
- By analyzing the interactions between electrolyte chemicals at the molecular level, we explored what physical properties are involved in the interactions.

## Results

**Figure 1:**
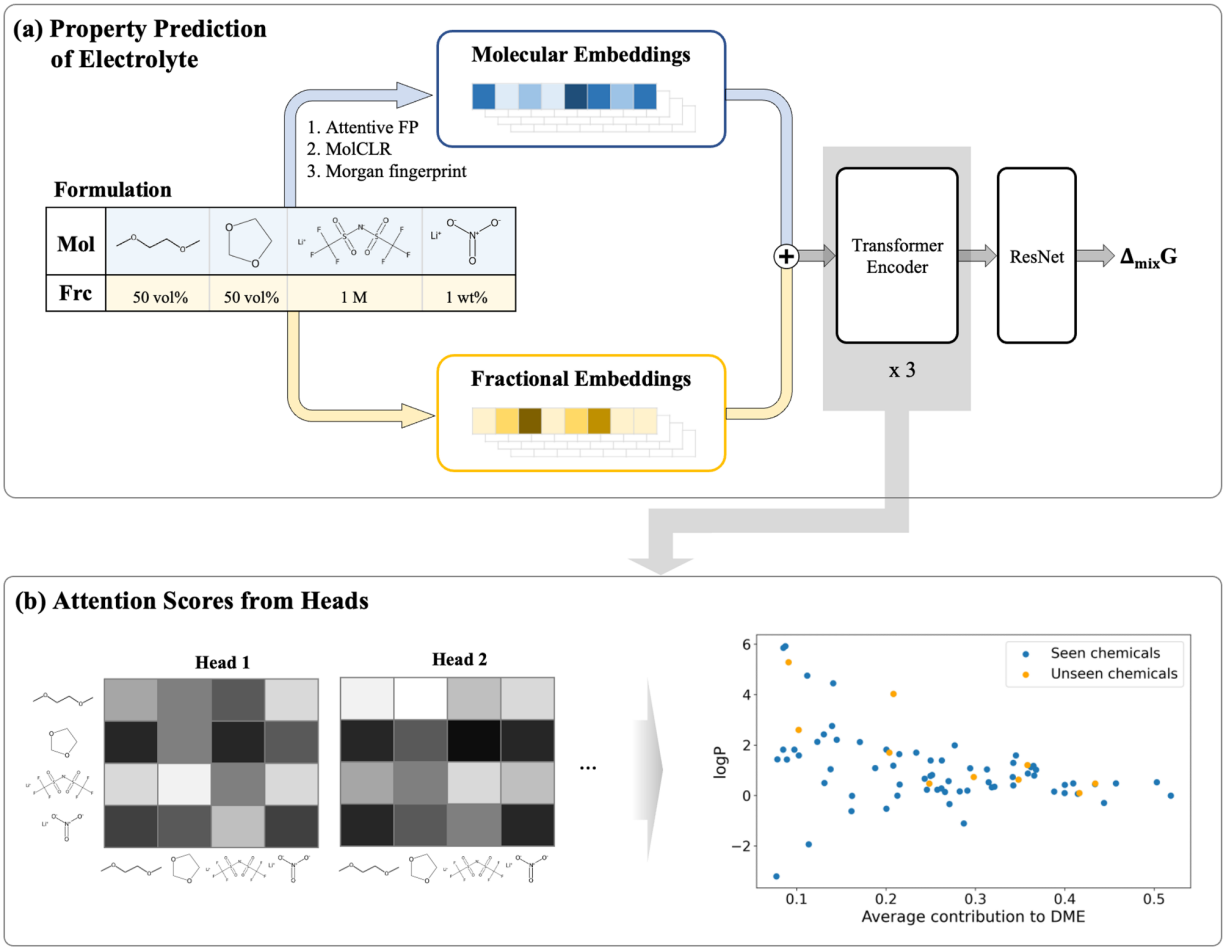
$\Delta _{mix}\textrm{G}$ Prediction framework and average contributions of chemicals attributing to others and how train the architectures. (**a**) Overall framework is drawn. A formulation consists of combinations of molecules and their ratios. Molecular encoding (Attentive FP, MolCLR and Morgan fingerprint) and fractional encoding are applied to each molecules (Mol) and fractions (Frc). And summations of the molecular embeddings and the fractional embeddings go through the three transformer encoder layers and ResNet^11^ network to get $\Delta _{mix}\textrm{G}$ values. (**b**) The attention values from multi-head attention layers of last transformer encoder layer could be visualized and further analyses were conducted in the molecular wise.

The overall outline of this study consisting of three parts is shown in Fig. 1. First, frameworks to predict $\Delta _{mix}\textrm{G}$ of electrolyte formulations were designed and the best predictive model was selected. Subsequently, the attention scores were analyzed to identify the origins of intermolecular interactions.

### $\Delta _{mix}\textrm{G}$ prediction framework

We compared frameworks to find the best way to predict $\Delta _{mix}\textrm{G}$ of electrolyte formulations with 100k of simulation data. The frameworks were designed by combining three molecular feature extraction representations(Morgan Fingerprint(MF)^12^, MolCLR(MCR)^13^, Attentive FP(AF)^10^) with two regressors(simple Multi Layer Perceptrons(MLP), Transformer Encoder(TE)^14^). More details of data, representations and regressors are written in the method section. In Table 1, L1 loss(MAE) of six architectures were compared to get the best one. The architectures employing TE demonstrated good predictive performances, with loss differences less than 0.01. This indicates that the transformer encoder effectively encodes formulation information, resulting in excellent performance regardless of the feature extractions. Parity plots of each architecture can be seen in the supplementary Fig. S1.

**Table 1:** Evaluation: Train/Val/Test set L1 loss(R2 score).

Representation	Regressor	Train loss (R2 score)	Val loss (R2 score)	Test loss (R2 score)
MF	MLP	0.041 (0.996)	0.042 (0.996)	0.042 (0.996)
MCR	MLP	0.312 (0.824)	0.313 (0.823)	0.312 (0.824)
AF	MLP	0.086 (0.987)	0.087 (0.986)	0.086 (0.962)
**MF**	**TE**	**0.005 (1.000)**	**0.008 (1.000)**	**0.008 (1.000)**
MCR	TE	0.013 (1.000)	0.014 (1.000)	0.014 (1.000)
AF	TE	0.007 (1.000)	0.010 (1.000)	0.010 (1.000)

The method that achieved the best performance and the corresponding losses and R2 scores have been highlighted in bold.

However, when conducting electrolyte researches, researchers test new chemicals in addition to the conventional chemicals in our dataset, so the model can be practically used when it can predict the properties of electrolytes containing new chemicals well. To evaluate the model in this respect, we divided the 69 solvents into seven groups, each of which has 10, 10, 10, 10 ,10, 10, and 9 solvents. The list of 69 solvents can be found in the supplementary information. For the seven cases where formulations containing solvents from each group were designated as the test set, we trained the models by taking each of the remaining six groups as the validation set and the rest as the training set. Thus, we checked the performance of each model on a total of 42 cases, and the average L1 losses and R2 scores are recorded in Table 2. Details on the multiple 6-fold cross-validation method can be found in the method section and Fig. 6. The transformer encoder with Attentive FP has the lowest loss compared to other architectures, which is more pronounced than the difference in Table 1. With a mean R2 score of 0.835 and the smallest standard deviation, it performed well in predicting electrolytes containing chemicals not seen during training. As a result, this was chosen as the most suitable architecture for predicting $\Delta _{mix}\textrm{G}$, and further analyses were conducted using this model.

**Table 2:** Evaluation for unseen chemicals during training: Mean and standard deviation of L1 losses and R2 scores of 42 cases (6 validation sets were used within the 7 test sets for each model).

Representation	Regressor	Mean (standard deviation) of test losses	Mean (standard deviation) of test R2 scores
MF	MLP	0.499 (0.103)	0.468 (0.193)
MCR	MLP	0.668 (0.086)	0.209 (0.231)
AF	MLP	0.275 (0.115)	0.806 (0.156)
MF	TE	0.336 (0.106)	0.732 (0.149)
MCR	TE	0.566 (0.098)	0.354 (0.225)
**AF**	**TE**	**0.230 (0.092)**	**0.835 (0.114)**

The method that achieved the best performance and the corresponding losses and R2 scores have been highlighted in bold.

### Intermolecular attention map analysis

The transformer encoder not only showed good performance in predicting $\Delta _{mix}\textrm{G}$ but also provided an explanation of which interaction is important in predicting $\Delta _{mix}\textrm{G}$ between the chemicals of the electrolytes through attention scores. These attention scores here can be regarded as interactions between the chemicals in $\Delta _{mix}\textrm{G}$ prediction. An attention map was obtained from a last transformer encoder layer, consisting of 4 head attentions, which allows us to visualize the degree of interactions between the chemicals.

**Figure 2:**
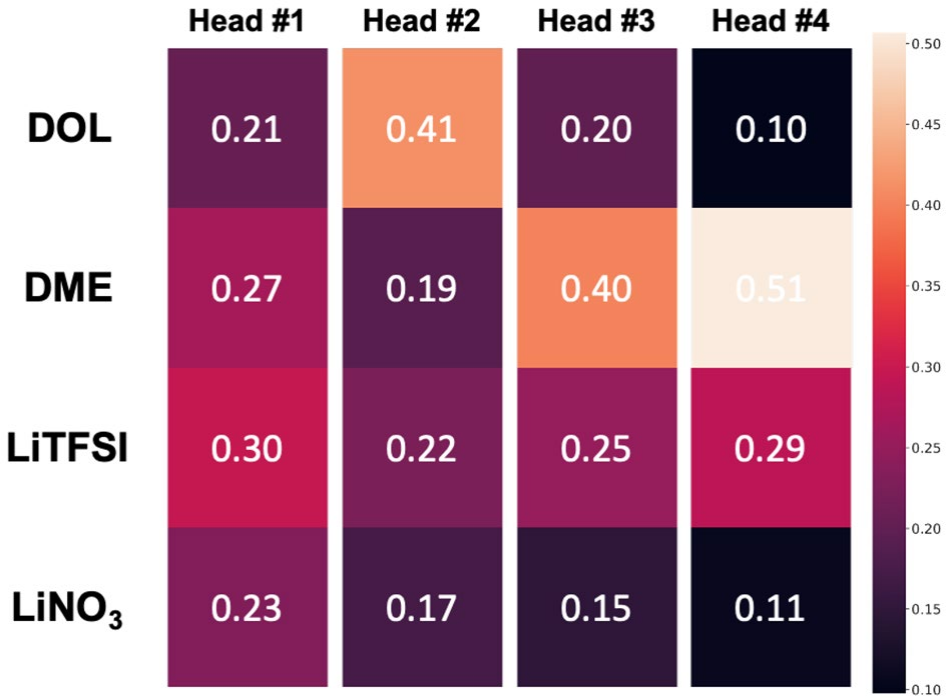
Average contributions of chemicals attributing to others in specific formulations consisting of DOL, DME, LiTFSI, LiNO3. The average contributions of 1,3-dioxolane (DOL), 1,2-dimethoxyethane (DME), lithium;bis(trifluoromethylsulfonyl)azanide(LiTFSI), and lithium;nitrate (LiNO_3_) to others, which are representative chemicals of electrolytes for the lithuim-sulfur batteries, were compared. The lighter the color, the greater the contribution. Average contributions were obtained from each attention head of last transformer encoder layer and are shown.

For example, attention scores were examined on common electrolytes in lithium-sulfur batteries such as 1,3-dioxolane (DOL), 1,2-dimethoxyethane (DME), lithium;bis(trifluoromethylsulfonyl)azanide (LiTFSI) and lithium;nitrate (LiNO3). The average of attention scores from 1881 formulations generated by grid search were used. In the Fig. 2, the contributions of each chemical to the others were compared by visualizing the heat maps and the contributions to the other chemicals were different in each head (#1~#4). In the first head (#1), four components are important overall. DOL is the most important component in the second (#2), and DME is in the third (#3) and fourth (#4). From this, each attention head has different points of views regarding important and DME affects other components more than other chemicals.

In order to obtain practical perspective, further attention analysis was conducted by comparing attention values of chemicals dedicating to conventional solvent 1,2-dimethoxyethane (DME). DME is a primary component of commonly used electrolytes due to its high performance in stabilizing a lithium metal anode during charge/discharge cycles. However, its high polysulfide solubility leads to the shuttling effect, prompting researches to explore new co-solvent candidates which can reduce the solubility of LiPS without decreasing electrolyte’s ionic conductivity^15,16^. While our analysis did not consider the ionic conductivity of the electrolyte, it explained the effects of co-solvents on polysulfide solubility through attention values. To investigate the effects of co-solvents, the degree to which DME refers to other chemicals can be obtained from the self-attention map, which can be understood as how much other chemicals affect DME. The five highest and five lowest contributors to DME were compared to find commonalities, and contributions to DME of each chemical were correlated with the properties of the chemicals to determine which properties were associated with these. Since the average attention scores vary depending on the number of chemicals in the electrolyte when extracting attention scores, we used only electrolytes composed of 6 chemicals (48k out of 100k data). In the supplementary information, chemicals are listed in decreasing order of average attention values from all heads contributing to the DME.

**Figure 3:**
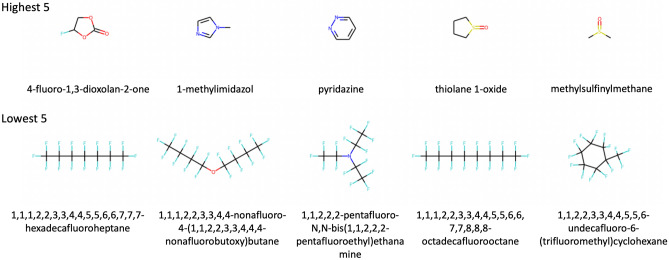
Each of the five chemicals with the highest and lowest average contributions to DME. Carbonate and imidazole and sulfoxide which make carbon electrophilic are found in the highest five chemicals. On the other hand, five chemicals with the lowest contribution have fluorine groups.

**Figure 4:**
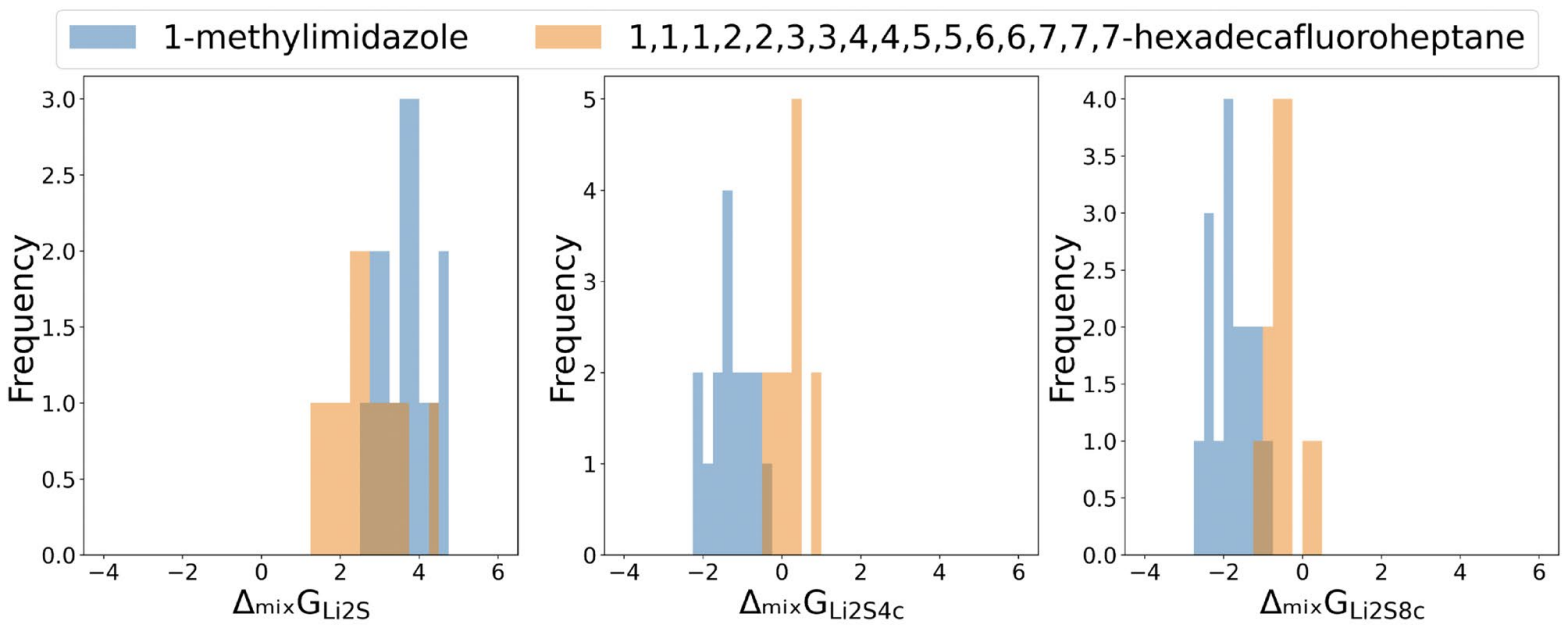
The comparison of the distribution of $\Delta _{mix} \textrm{G}$. We compared the data distribution of $\Delta _{mix}\textrm{G}$ when the chemical with the second highest contribution chemical (1-methylimidazol) and the chemical with the lowest contribution(1,1,1,2,2,3,3,4,4-nonafluoro-4-(1,1,2,2,3,3,4,4,4-nonafluorobutoxy)butane) constituting the electrolyte with DME. When DME was combined with the second highest chemical, the distribution of long-chain $\Delta _{mix}\textrm{G}$ values were lower than when it was combined with the lowest one, indicating that solubility of polysulfides is larger.

The top five chemicals with the highest contributions and the five with the lowest can be seen in the Fig. 3. The five highest chemicals include carbonate, imidazole and sulfoxide which have high reactivity because they induce carbons electrophilic. Especially, it is well known that the electrophilic organic carbonates are reactive toward the nucleophilic lithium polysulfides and consistent with a previous study^17^. And it has been reported that 1-methylimidazole can dissolve $Li_2S_6$ well in a previous study^18^. On the other hand, five chemicals with the lowest contribution have fluorine groups. Having a fluorine group results in very low reactivity due to the stability of the C-F bond. A previous study^19^ suggested that fluorinated ethers have lower solubility than general ethers because the fluorine has more electronegativity and stronger steric hindrance than the hydrogen. Based on the five highest and lowest contributing chemicals, it can be inferred that contributions are related to the solubility of polysulfides. We compared data distributions of $\Delta _{mix}\textrm{G}$ when the chemicals with the second highest contribution chemical(1-methylimidazol) which is known to dissolve polysulfide well and the chemicals with the lowest contribution(1,1,1,2,2,3,3,4,4,5,5,6,6,7,7,7-hexadecafluoroheptane) constituting the electrolyte with DME in Fig. 4. As shown in this, in general, when DME was combined with the second highest chemicals, the distribution of long-chain $\Delta _{mix}\textrm{G}$ values were lower than when it was combined with the lowest one, indicating that the solubility of polysulfides is larger. Therefore, it can be regarded that the self-attention can capture the interactions between chemicals that are important in predicting $\Delta _{mix}\textrm{G}$. The distribution comparison with the other highest chemicals and the lowest one can be in the Fig. S2. In addition, we analyzed the contribution to DME as well as other solvents(1,1,1,2,2,3,3,4,4,5,5,6,6,7,7,7-hexadecafluoroheptane, tetramethylenesulfoxide). Similar to the case of DME, most of the chemicals with high contribution have polarity, and most of chemicals with low contribution have flourines. These chemicals can be seen in the supplementary Figs. S3 and S4.

**Figure 5:**
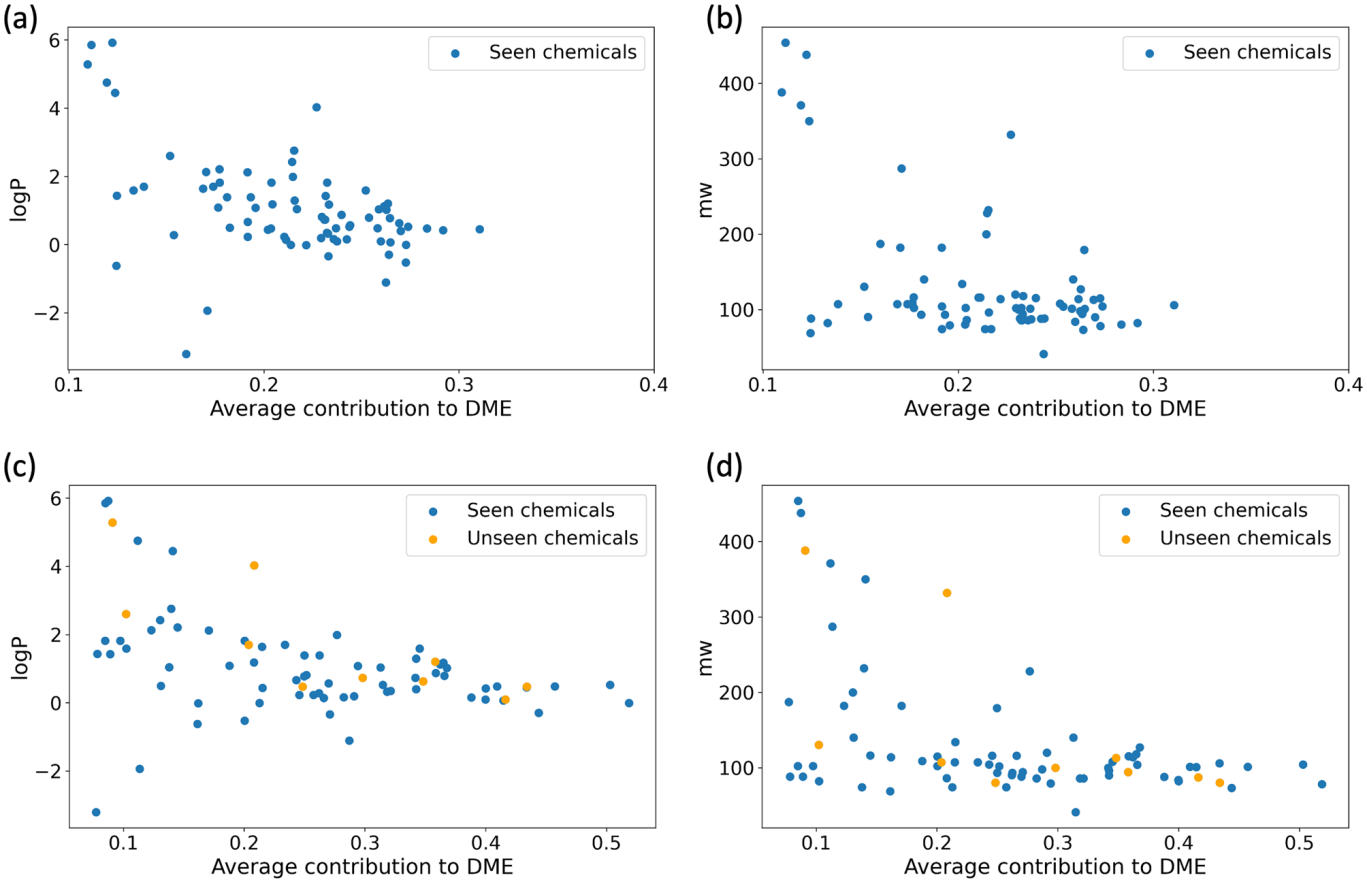
Scatter plots of average contribution to DME of chemicals versus logP and molecular weight. We investigated the relationship between physical properties and average contributions and visualized it using scatter plots. (**a**) and (**b**) show the average contribution to DME from the model when all chemicals are used in training and scatter plots against logP and molecular weight. The Pearson correlation coefficients are − 0.49 and − 0.50, respectively. In (**c**) and (**d**), the average contribution to DME was extracted from the model trained without some chemicals in training and scatter plotted against logP and molecular weight. The chemicals used in the training are colored blue and the chemicals not used are colored orange. The Pearson correlation coefficients are − 0.41 and − 0.47, respectively, and It was confirmed that chemicals not used in training also exhibit physical properties and tendencies similar to those of the chemicals that were used during training.

Additionally, we correlated how the contribution to DME, obtained from attention scores, relates with physical properties. These properties include logP, molecular weight, atomic polarizability, bond polarizability, and the smr (Wildman–Crippen MR descriptor) which are highly related to the solubility. Among several properties, logP and molecular weight have pearson correlation coefficients of $-0.49$ and $-0.50$ respectively, suggesting a negative correlation (Fig. 5a,b). And the correlation analysis with other properties can be seen in the supplementary Fig. S5. In the previous study^20^ that developed novel methods for predicting logP value using diverse molecular structures, it was found that logP highly correlates with molecular polarizability and partial atomic charges on nitrogen and oxygen, with a correlation coefficient of 0.89. And molecular weight affecting the size of the molecular size, is also related to the molecular polarizability. From these, we consider that the self-attention heads can capture the chemical property(polarizability) related to $\Delta _{mix}\textrm{G}$. Scatter plots of other properties versus contributions were drawn in the Fig. S5. To ascertain if the correlation analysis results with physical features would remain consistent for chemicals not used during training, we used a model trained without certain chemicals in the training to extract the contribution of chemicals to DME and analyze their correlation with physical attributes. In line with the analysis using the model trained with all chemicals, there was a pearson correlation of – 0.4 between contribution and logP, and – 0.47 between contribution and molecular weight. Interestingly, the chemicals that were excluded from the training demonstrated trends similar to those that were included in the training, as illustrated in Fig. 5.

## Discussion

We developed a framework to predict $\Delta _{mix}\textrm{G}$, a crucial property for LiS batteries, for formulations with various chemicals and different fractions. The transformer encoder with representations demonstrated high predictive performances, with R2 scores of approximately 1.0, and the representation from Attentive FP provided the best generalizability on unseen chemicals, with average test R2 scores of 0.835. By it can predict properties of formulations that made of not only chemicals used in training, but also chemicals not included in the train dataset well. Furthermore, we conducted intermolecular attention map analyses and found that the average contributions to DME correlated with logP and molecular weight related to molecular polarizability. We also identified specific functional groups in the top and bottom five contributing chemicals, confirming that the attention layers are able to capture distinct molecular features. Our framework can be applied to various promising directions especially for material combinations in formulations. In the real world, useful products are mainly combinations of different materials rather than single materials, which makes our architecture more useful. However, there are few limitations on our approach. Firstly, the our model doesn’t consider the environment conditions such as temperature. Secondly, this framework assumes that chemical components stay in same which means there is no chemical reaction. Additionally, the model will have low prediction performance on a mixture including a chemical which is very different to chemicals in molport database. In the future work, those three limitations should be addressed to make the model more practical.

## Methods

### Data preparation and generation

The electrolyte formulation $\Delta _{mix}\textrm{G}$ data was generated through Conductor-like Screening Model for Real Solvents (COSMO-RS), considered as the most accurate model for estimating solvation energies^21,22^. The chemical potential, $\mu$, was yielded by calculating the charge distribution on the molecular surface based on the structure of the solvent and LiPS molecule, using the COSMOtherm softwares—COSMOlogic GmbH & Co. KG and TURBOMOLE. $\Delta _{mix}\textrm{G}$ is defined as $\Sigma \mu _j\nu _j$ (where $\mu _j$ is a chemical potential of substance j and $\nu _j$ is a stoichiometric coefficient of species j). Using this definition, $\Delta _{mix}\textrm{G}$ of each formulation can be calculated and classified into 8 properties :$\Delta _{mix}\hbox {G}_{\textrm{Li2S}}$, $\Delta _{mix}\hbox {G}_{\textrm{Li2S2}}$, $\Delta _{mix}\hbox {G}_{\textrm{Li2S4}_{\textrm{C}}}$, $\Delta _{mix}\hbox {G}_{\textrm{Li2S4}_{\mathrm{\mathrm L}}}$, $\Delta _{mix}\hbox {G}_{\textrm{Li2S6}_{\textrm{C}}}$, $\Delta _{mix}\hbox {G}_{\textrm{Li2S6}_{\mathrm{\mathrm L}}}$, $\Delta _{mix}\hbox {G}_{\textrm{Li2S8}_{\textrm{C}}}$ and $\Delta _{mix}\hbox {G}_{\textrm{Li2S8}_{\mathrm{\mathrm L}}}$ (subscripts C and L denote cyclic and linear form, respectively). All calculations were conducted at 25 °C, 1atom, and under the liquid phase condition. We randomly generated 100k electrolyte formulations consisting of solvents, anti-solvents, salt and additive, and calculated the corresponding $\Delta _{mix}\textrm{G}$ by COSMO-RS calculation. A list of solvents, antisolvents, salts and additives used can be found in the supplementary information.

### Modules for prediction models

#### Attentive FP

Attentive FP^10^ is a graph neural network architecture for molecular representations that accurately extracts information from chemical structures and offers interpretability. It employs two steps of attention layers for atom and molecule embedding and has demonstrated high predictive performances on diverse datasets. The network learns non-local intramolecular interactions strongly related to specific properties, as supported by feature visualization. Therefore, we pretrained Attractive FP using the values of apol, bpol, slogp, smr related to $\Delta _{mix}\textrm{G}$ value and extracted features from this pretrained model. Apol and bpol represent atom and bond polarizability, and slogp and smr are Wildman-Crippen LogP descriptors and MR descriptors, which are solubility related values. These four properties can be easily obtained using the mordred module^23^.

#### MolCLR

MolCLR^13^ is a framework that applies self-supervised graph neural network-based contrastive learning to molecules. First, it converts molecular smiles into a molecular graph. Atom masking, bond deletion, and subgraph removal are applied to these molecule graphs to create augmented graphs. In these graphs, the features that have undergone the convolutions and readout processes are trained to exhibit high similarity for features from the same molecule and low similarity for features from different molecules through contrastive loss, as employed in the previous study^24^. The biggest advantage of this framework is that this model can be pre-trained using a lot of data without additional labeling.

#### Morgan fingerprint

Morgan fingerprint^12^ is the one of the most popular molecular fingerprint. It encodes structural information of a molecule as a vector, bit strings and we used RDKit^25^ to convert a molecule from SMILES to the vector. Therefore, it is widely used in the field of new drug development, especially when finding similarities between molecules.

#### Multi layer perceptron

The multi layer perceptron^26^ is a type of artificial neural network composed of multiple layers and nodes, capable of learning complex non-linear patterns. It consists of an input layer, one or more hidden layers, and an output layer. Nodes in each layer are connected to the previous layer through weights and activation functions. In this study, molecular embeddings were multiplied by fractions of each component and used as input.

#### Transformer encoder

Transformer^14^, first introduced in the natural language processing field, has demonstrated high performance across various domains. Transformer encoder can flexibly encode words by considering the degree of influence between them depending on the context. To encode chemicals in each formulation, we used molcular embededding to represent each molecule and fractional encoding instead of positional encoding to represnet each fraction, as done in a previous study^27^. The rest of the model remains identical to the original one. Additionally, by analyzing multi-head attention scores, we were able to visualize interactions between components in each formulation.

### Training details

**Figure 6:**
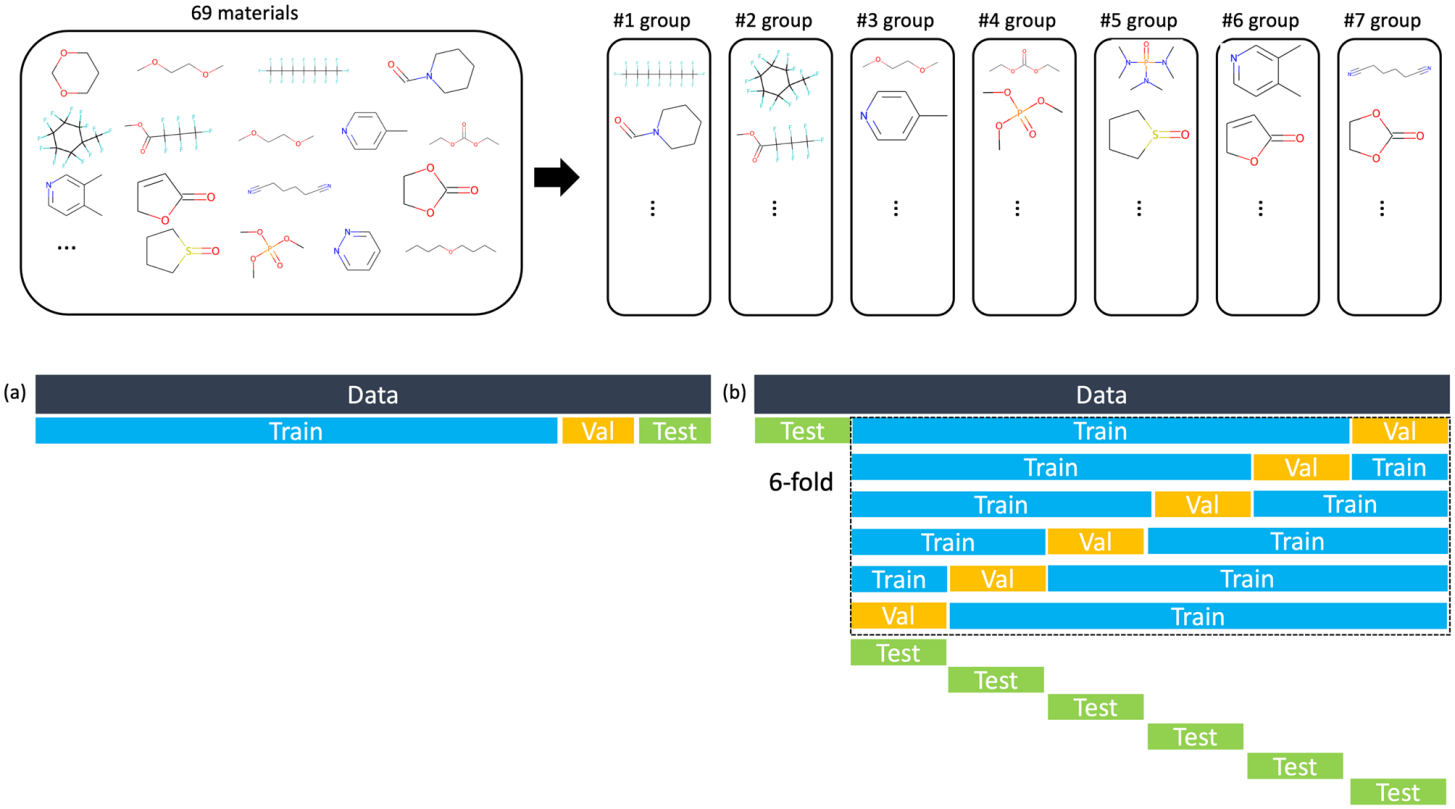
Training process We splitted chemicals existing in the data into 7 groups. (**a**) To compare general predictive performance, dataset was split with the ratio of train : valid : test at 8:1:1. (**b**) In order to compare the predictive power for unseen chemicals, each group was designated as a test set and 6-fold cross validations were conducted for each test set.

For a fair evaluation of the six architectures compared in the result part, training was conducted by setting the hyperparameters to be the same as possible. The dimension of embeddings used for training was set to 256. We used molport database^28^ to pretrain Attentive FP and MolCLR to get the embeddings of chemicals, which contains 480 million molecules. The formulation dataset was split with the ratio of train: valid: test at 8:1:1 Fig. 6a. The batch size was set to 16384 to speed up training, and dropout was 0.1 to avoid overfitting. The training results of six architectures can be seen in Table 1 and supplementary Fig. S1. To evaluate the model’s predictive performance for unseen chemicals during training, the data was divided into a 7-fold cross-validation setup. Out of the 77 chemicals in the dataset, 69 chemicals (excluding 3 types of salts and additives that are always used, and 5 types of frequently used solvents) were divided into 7 groups. Six-fold cross-validations were conducted, using each of the 7 groups as the test set as seen in Figure 6b. In other words, training was carried out for 42 cases per architecture, and the predictive performances were compared with the average of the losses. The mean and standard deviation of the losses are recorded in Table 2. The parameters used during training were same as when training was conducted on the entire data.

### Supplementary Information

Supplementary Information.

## Data Availability

The data that support the findings of this study is available from LG Energy Solution. but restrictions apply to the availability of these data, which were used under license for the current study, and so are not publicly available. Data are however available from the authors upon reasonable request and with permission of LG Energy Solution. Requests for the data can be made by emailing one of the authors, Seong-Hyo Park(seonghyopark@lgensol.com).
