# Supplementary material for: Attention-based solubility prediction of polysulfide and electrolyte analysis for lithium–sulfur batteries
Source: Sci Rep. 2023 Nov 27;13:20784. doi: 10.1038/s41598-023-47154-0 (PMC10682475; doi:10.1038/s41598-023-47154-0)
Supplement: Supplementary file 1 — Supplementary Information. [file 41598_2023_47154_MOESM1_ESM.pdf]

Supplementary information

A list of chemicals used to generated data

Solvents(69):

methylsulfonylmethane
1,1,3,3-tetramethylurea
1,1,1,2,2,3,3,4,4-nonafluoro-4-(1,1,2,2,3,3,4,4,4-nonafluorobutoxy)butane)
N,N-dimethylnitrous amide
1,1,1,3,3,3-hexafluoro-2-methoxypropane
trimethyl phosphate
1-methoxyhexane
1-methylimidazole
4-methoxypyridine
piperidine-1-carbaldehyde
pyridine
4-methylpyridine
oxolan-2-one
3,4-dimethylpyridine
1-ethoxy-2-methoxyethane
3-methyl-1,3-oxazolidin-2-one
3-methylpyridine
N,N-dimethylbutanamide
butanedial
3,5-dimethylpyridine
2,4-dimethoxypyrimidine
dimethyl carbonate
hexanedinitrile
ethoxyethane
1-propoxypropane
1,1,2,2,3,3,4,4-octafluoro-5-(1,1,2,2-tetrafluoroethoxy)pentane
N,N-dimethylacetamide
1,3-dimethylimidazolidin-2-one
pyridazine
1,3-dioxolane
N,N-diethylformamide
methyl prop-2-enoate
1,1,2,2,3,3,4,4,5,5,6-undecafluoro-6-(trifluoromethyl)cyclohexane)
diethyl carbonate
methylsulfinylmethane
methyl 2-methylpropanoate
methyl 2,2,3,3,4,4,4-heptafluorobutanoate
methyl propanoate
methyl 3-methylbut-2-enoate
1,3-dioxolan-2-one
2H-furan-5-one
cyclohex-2-en-1-one
1,1,1,2,2,3,3,4,4,5,5,6,6,7,7,7-hexadecafluoroheptane
morpholine-4-carbaldehyde
4-ethylpyridine
N-[bis(dimethylamino)phosphoryl]-N-methylmethanamine
acetonitrile
thiolane 1,1-dioxide
2-methyloxolane
1-piperidin-1-ylethanone
2-ethoxy-2-methylpropane

```
methyl but-2-enoate
thiolane 1-oxide
4-fluoro-1,3-dioxolan-2-one
1,1,1,2,2,3,3,4,4,5,5,6,6,7,7,8,8,8-octadecafluorooctane
1,1,2,2,2-pentafluoro-N,N-bis(1,1,2,2,2-pentafluoroethyl)ethanamine
pentanedinitrile
pyrimidine
methyl 2-methylprop-2-enoate
ethyl methyl carbonate
1,2-dimethoxyethane
2-methoxy-2-methylpropane
2-propan-2-yloxypropane
3-methylthiolane 1,1-dioxide
N,N-dimethylpropanamide
1-butoxybutane
methyl 2-cyanoacetate
methyl 3-oxobutanoate
N,N-dimethylformamide
```

Anti Solvents(5):

```
1,1,2,2-tetrafluoro-1-(2,2,2-trifluoroethoxy)ethane
1,1,2,2-tetrafluoro-3-(1,1,2,2-tetrafluoroethoxy)propane
2-methylfuran
1,1,1-trifluoro-2-(2,2,2-trifluoroethoxy)ethane
1-ethoxypropane
```

Salts(2):

```
lithium;bis(trifluoromethylsulfonyl)azanide
lithium;bis(fluorosulfonyl)azanide
```

Additive(1):

```
lithium;nitrate
```

Supplementary Table S1. The average contributions of materials to DME

Materials	average contribution score to DME
4-fluoro-1,3-dioxolan-2-one	0.311
1-methylimidazole	0.292
pyridazine	0.284
thiolane 1-oxide	0.274
methylsulfinylmethane	0.273
morpholine-4-carbaldehyde	0.273
dimethyl carbonate	0.270
piperidine-1-carbaldehyde	0.269
3-methyl-1,3-oxazolidin-2-one	0.265
Hexamethylphosphoramide	0.265
N-[bis(dimethylamino)phosphoryl]-N-methylmethanamine	0.264
pentanedinitrile	0.263
1-piperidin-1-ylethanone	0.263
methyl 2-cyanoacetate	0.262
methyl 3-methylbut-2-enoate	0.262
2H-furan-5-one	0.260

trimethyl phosphate	0.259
N,N-diethylformamide	0.258
ethyl methyl carbonate	0.254
hexanedinitrile	0.252
methyl propanoate	0.244
acetonitrile	0.244
1,3-dioxolan-2-one	0.242
N,N-dimethylbutanamide	0.239
N,N-dimethylacetamide	0.237
N,N-dimethylpropanamide	0.2369
butanedial	0.235
diethyl carbonate	0.233
methylsulfonylmethane	0.232
oxolan-2-one	0.232
1-propoxypropane	0.232
methyl prop-2-enoate	0.232
2-methoxy-2-methylpropane	0.232
methyl 2-methylprop-2-enoate	0.231
methyl but-2-enoate	0.231
methyl 2-methylpropanoate	0.230
thiolane 1,1-dioxide	0.229
1,1,2,2,3,3,4,4-octafluoro-5-(1,1,2,2-tetrafluoroethoxy)pentane	0.227
1,3-dimethylimidazolidin-2-one	0.222
ethoxyethane	0.217
cyclohex-2-en-1-one	0.216
1,1,2,2-tetrafluoro-3-(1,1,2,2-tetrafluoroethoxy)propane	0.215
methyl 2,2,3,3,4,4,4-heptafluorobutanoate	0.215
1,1,2,2-tetrafluoro-1-(2,2,2-trifluoroethoxy)ethaner	0.214
1,3-dioxolane	0.214
methyl 3-oxobutanoate	0.211
1,1,3,3-tetramethylurea	0.2104
2-methyloxolane	0.204
2-ethoxy-2-methylpropane	0.2037
pyrimidine	0.2034
3-methylthiolane 1,1-dioxide	0.2019
pyridine	0.196
3-methylpyridine	0.193
N,N-dimethylnitrous amide	0.192
1-ethoxy-2-methoxyethane	0.192
1,1,1,3,3,3-hexafluoro-2-methoxypropane	0.192
2,4-dimethoxypyrimidine	0.183
4-methylpyridine	0.181
2-propan-2-yloxypropane	0.177
1-methoxyhexane	0.177
4-methoxypyridine	0.177
3,4-dimethylpyridine	0.174
lithium;bis(trifluoromethylsulfonyl)azanide	0.171
1,1,1-trifluoro-2-(2,2,2-trifluoroethoxy)ethane	0.170
4-ethylpyridine	0.169
lithium;bis(fluorosulfonyl)azanide	0.160
1,2-dimethoxyethane	0.154
1-butoxybutane	0.152
3,5-dimethylpyridine	0.138
2-methylfuran	0.133
1-ethoxypropane	0.125

lithium;nitrate	0.124
1,1,2,2,3,3,4,4,5,5,6-undecafluoro-6-(trifluoromethyl)cyclohexane	0.124
1,1,1,2,2,3,3,4,4,5,5,6,6,7,7,8,8,8-octadecafluorooctane	0.122
1,1,2,2,2-pentafluoro-N,N-bis(1,1,2,2,2-pentafluoroethyl)ethanamine	0.119
1,1,1,2,2,3,3,4,4-nonafluoro-4-(1,1,2,2,3,3,4,4,4-nonafluorobutoxy)butane	0.111
1,1,1,2,2,3,3,4,4,5,5,6,6,7,7,7-hexadecafluoroheptane	0.110

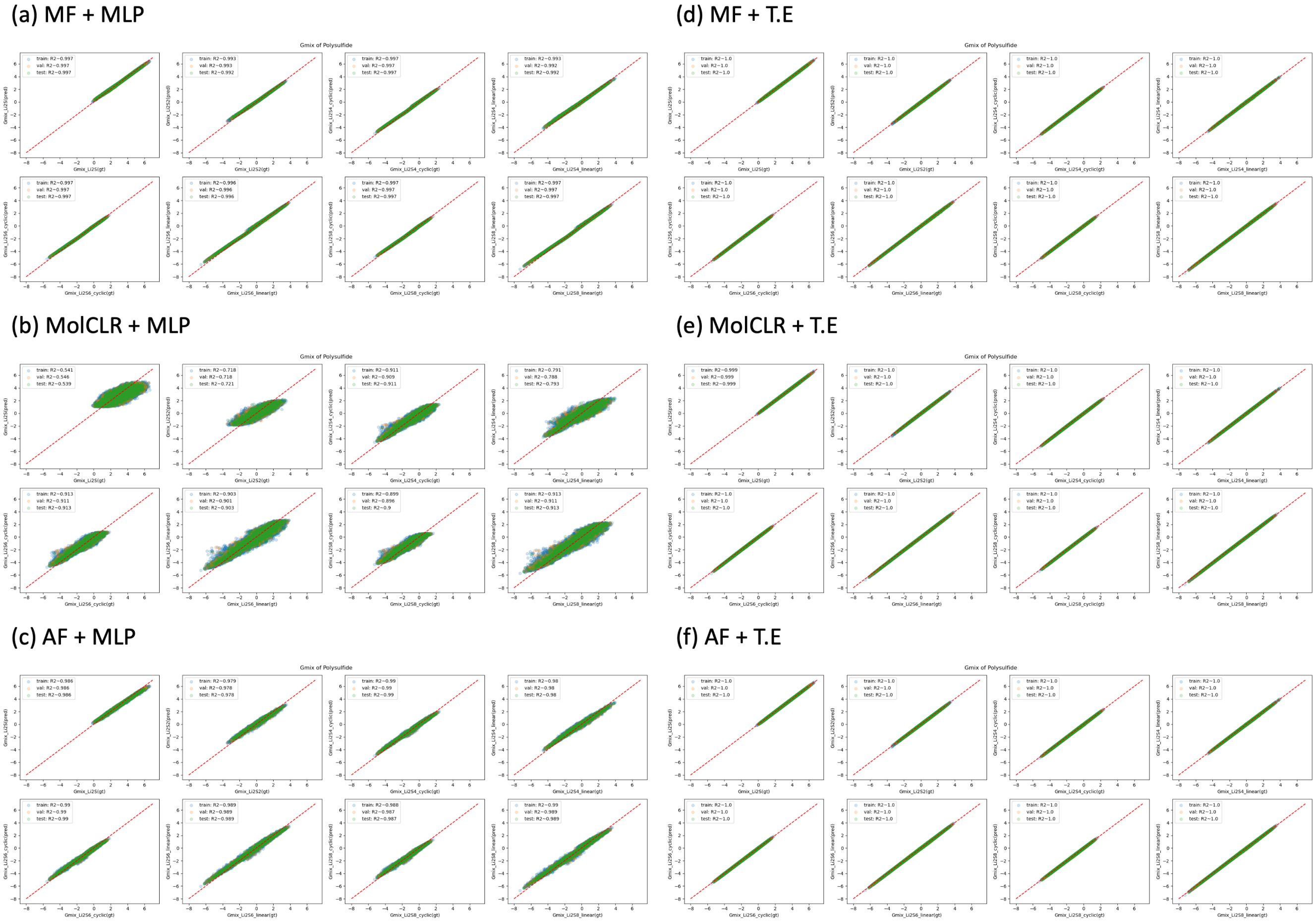

Supplementary figure S1. Parity plots of six architectures (1) MF+MLP, (2) MCR+MLP, (3) AF+MLP (4) MF+T.E (5) MCR+T.E (6) AF+T.E (MF, MCR, AF, MLP and T.E denotes Morgan Fingerprint, MolCLR, Attentive Fingerprint, Transformer Encoder, respectively). Transformer encoder improved the predictive performance regardless of embeddings. The predictive performances of MF+T.E, MCR+T.E, AF+T.E are good and almost same.

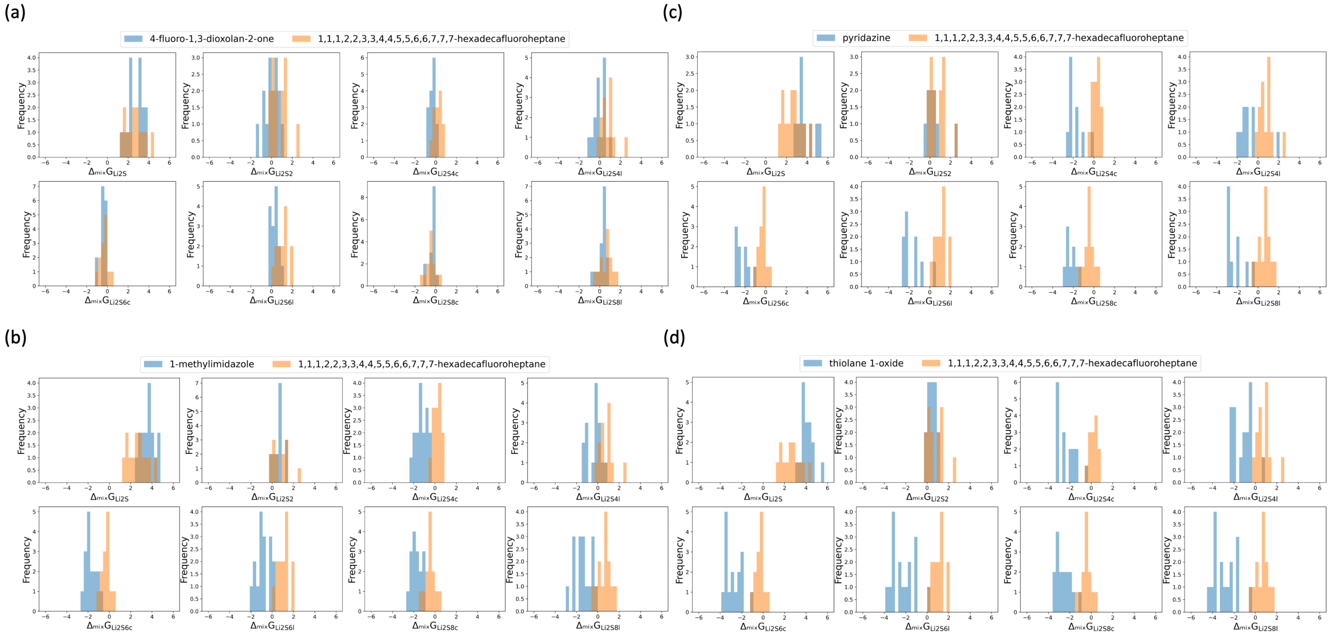

Supplementary figure S2. The comparison of the distribution of ΔG_{mix}. We compared the data distribution of ΔG_{mix} when the material with the highest contribution materials(4-fluoro-1,3-dioxolan-2-one, 1-methylimidazol, pyridazine, thiolane 1-oxide) and the material with the lowest contribution(1,1,1,2,2,3,3,4,4,5,5,6,6,7,7,7-hexadecafluoroheptane) constituting the electrolyte with DME in figure. When DME was combined with the highest materials, the distribution of long-chain ΔG_{mix} values were lower than when it was combined with the lowest one, indicating that solubility of polysulfides is large except (a).

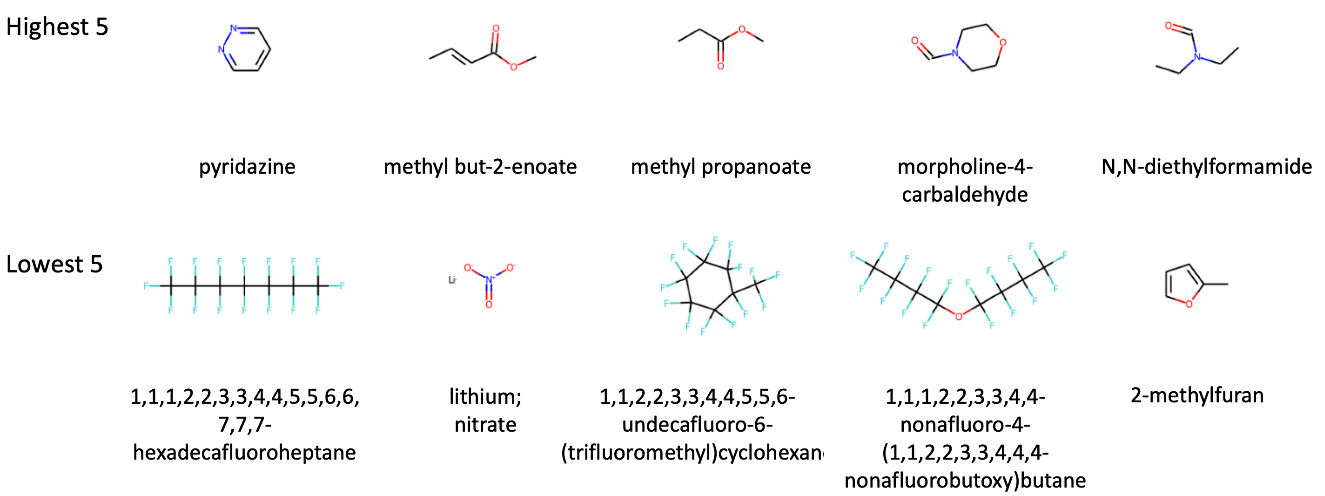

Supplementary figure S3. Each of the five materials with the highest and lowest average contributions to 1,1,1,2,2,3,3,4,4,5,5,6,6,7,7,7-hexadecafluoroheptane. Polar materials are found in the highest five materials. On the other hand, five materials with the lowest contribution have fluorine groups.

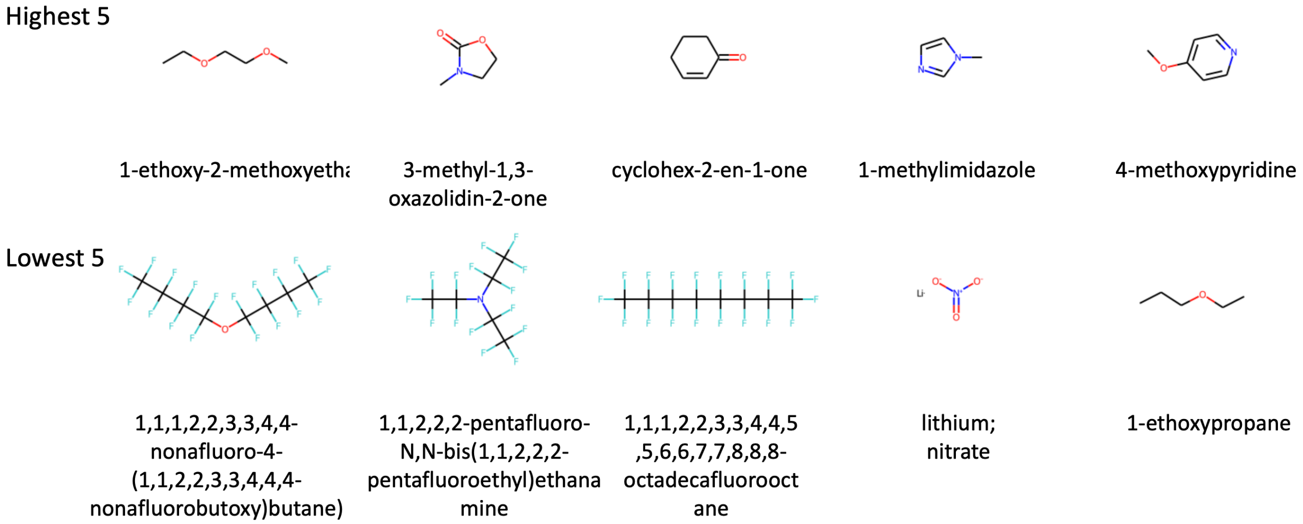

Supplementary figure S4. Each of the five materials with the highest and lowest average contributions to thiolane 1-oxide. Polar materials are found in the highest five materials. On the other hand, five materials with the lowest contribution have fluorine groups.

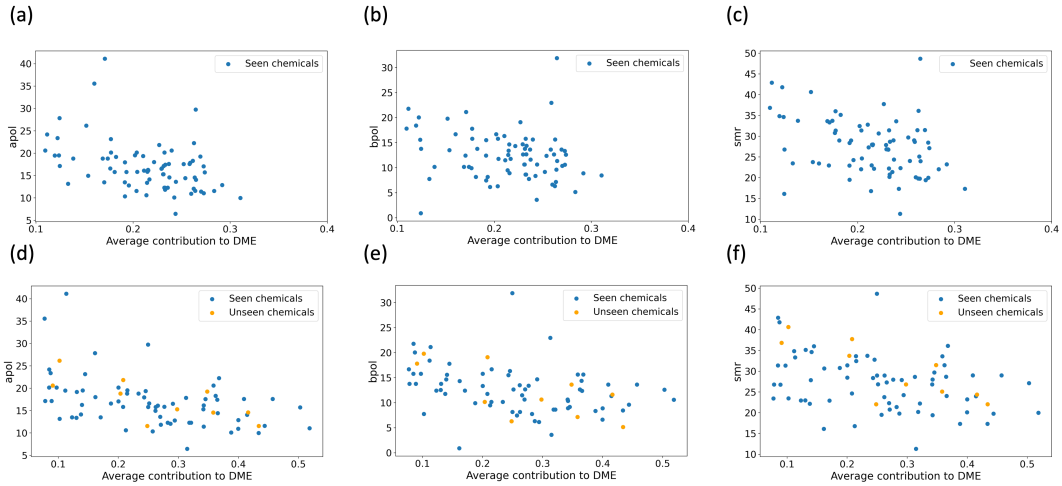

Supplementary figure S5. Scatter plots of average chemical contributions to DME versus other physical properties Scatter plots were drawn when the x-axis is contribution to DME and the y-axis is molecular weight, atom polarizability, bond polarizability and smr, respectively. In the (a),(b) and (c), contributions to DME is extracted from the model trained with all chemicals. The respective pearson correlation values versus molecular weight, atom polarizability, bond polarizability and smr are -0.5, -0.42, -0.20, and -0.36. On the other hand, in the (d),(e) and (f), contributions to DME is extracted from the model trained with some chemicals. During the training, seen chemicals and unseen chemicals are denoted as blue dots and oragne dots, respectively. The respective pearson correlation values versus molecular weight, atom polarizability, bond polarizability and smr are -0.47, -0.47, -0.36, and -0.37.
